# XENERA-1: a randomised double-blind Phase II trial of xentuzumab in combination with everolimus and exemestane versus everolimus and exemestane in patients with hormone receptor-positive/HER2-negative metastatic breast cancer and non-visceral disease

**DOI:** 10.1186/s13058-023-01649-w

**Published:** 2023-06-12

**Authors:** Peter Schmid, Javier Cortes, Ana Joaquim, Noelia Martínez Jañez, Serafín Morales, Tamara Díaz-Redondo, Sibel Blau, Patrick Neven, Julie Lemieux, José Ángel García-Sáenz, Lowell Hart, Tsvetan Biyukov, Navid Baktash, Dan Massey, Howard A. Burris, Hope S. Rugo

**Affiliations:** 1 Centre for Experimental Cancer Medicine, Barts Cancer Institute, Queen Mary University of London, London, UK; 2 International Breast Cancer Center (IBCC), Pangaea Oncology, Quironsalud Group, Barcelona, Spain; 3 Faculty of Biomedical and Health Sciences, Department of Medicine, Universidad Europea de Madrid, Madrid, Spain; 4 Centro Hospitalar de Vila Nova de Gaia/Espinho, Vila Nova de Gaia, Portugal; 5 Ramon y Cajal University Hospital, Madrid, Spain; 6 Hospital Arnau de Vilanova, Lleida, Spain; 7 Hospitales Universitarios Regional y Virgen de la Victoria de Málaga, Unidad de Gestión Clínica Intercentros de Oncología, Málaga, Spain; 8 Northwest Medical Specialties, Tacoma, WA USA; 9 UZ Leuven, Leuven, Belgium; 10 Centre Hospitalier Universitaire de Québec-Université Laval Research Centre, Quebec, Canada; 11 Hospital Clínico San Carlos, Madrid, Spain; 12 Florida Cancer Specialists, Fort Myers, FL USA; 13 Boehringer Ingelheim International GmbH, Ingelheim, Germany; 14 Boehringer Ingelheim (Canada) Ltd, Burlington, ON Canada; 15 Elderbrook Solutions GmbH on behalf of Boehringer Ingelheim Pharma GmbH & Co. KG, Biberach, Germany; 16 Sarah Cannon Research Institute, Nashville, TN USA; 17 University of California at San Francisco, San Francisco, CA USA

**Keywords:** Advanced breast cancer, HR+/HER2−, Non-visceral disease, Xentuzumab, Insulin-like growth factor, Everolimus, Exemestane

## Abstract

**Background:**

Xentuzumab is a humanised monoclonal antibody that binds to IGF-1 and IGF-2, neutralising their proliferative activity and restoring inhibition of AKT by everolimus. This study evaluated the addition of xentuzumab to everolimus and exemestane in patients with advanced breast cancer with non-visceral disease.

**Methods:**

This double-blind, randomised, Phase II study was undertaken in female patients with hormone-receptor (HR)-positive/human epidermal growth factor 2 (HER2)-negative advanced breast cancer with non-visceral disease who had received prior endocrine therapy with or without CDK4/6 inhibitors. Patients received a weekly intravenous infusion of xentuzumab (1000 mg) or placebo in combination with everolimus (10 mg/day orally) and exemestane (25 mg/day orally). The primary endpoint was progression-free survival (PFS) per independent review.

**Results:**

A total of 103 patients were randomised and 101 were treated (n = 50 in the xentuzumab arm and n = 51 in the placebo arm). The trial was unblinded early due to high rates of discordance between independent and investigator assessment of PFS. Per independent assessment, median PFS was 12.7 (95% CI 6.8–29.3) months with xentuzumab and 11.0 (7.7–19.5) months with placebo (hazard ratio 1.19; 95% CI 0.55–2.59; p = 0.6534). Per investigator assessment, median PFS was 7.4 (6.8–9.7) months with xentuzumab and 9.2 (5.6–14.4) months with placebo (hazard ratio 1.23; 95% CI 0.69–2.20; p = 0.4800). Tolerability was similar between the arms, with diarrhoea (33.3–56.0%), fatigue (33.3–44.0%) and headache (21.6–40.0%) being the most common treatment-emergent adverse events. The incidence of grade ≥ 3 hyperglycaemia was similar between the xentuzumab (2.0%) and placebo (5.9%) arms.

**Conclusions:**

While this study demonstrated that xentuzumab could be safely combined with everolimus and exemestane in patients with HR-positive/HER2-negative advanced breast cancer with non-visceral disease, there was no PFS benefit with the addition of xentuzumab.

*Trial registration* ClinicalTrials.gov, NCT03659136. Prospectively registered, September 6, 2018.

**Supplementary Information:**

The online version contains supplementary material available at 10.1186/s13058-023-01649-w.

## Background

Endocrine therapy has been the established treatment for patients with hormone receptor (HR)-positive, human epidermal growth factor receptor (HER2)-negative advanced breast cancer for many years. More recently, addition of cyclin-dependent kinase (CDK) 4/6 inhibitors to endocrine therapy has been shown to improve survival outcomes and is now considered standard of care in this setting [1]. Unfortunately, although most patients initially benefit, disease progression eventually occurs due to development of resistance. Subsequent treatment options are dependent on which agents were used in earlier settings, response to prior therapy and if specific mutations, such as *PIK3CA* mutations, are identified [1].

One option, based on findings of the Phase III BOLERO-2 trial, is exemestane plus everolimus, a mammalian target of rapamycin (mTOR) inhibitor [2]. While BOLERO-2 was conducted prior to the approval of CDK4/6 inhibitors, retrospective studies indicate that this combination is also effective following prior endocrine therapy plus a CDK4/6 inhibitor [3, 4].

Preclinical data suggest that the efficacy of mTOR inhibitors may be limited by compensatory signalling via the insulin-like growth factor (IGF) type 1 receptor (IGF-1R), which results in activation of AKT [5, 6]. Accordingly, inhibition of IGF signalling improved antitumour activity of mTOR inhibitors in xenograft models [5]. These findings provide rationale for assessing the combination of mTOR and IGF-1R inhibitors in the clinic. Moreover, as IGFs are considered to play a role in the development of bone metastases, it is possible that inhibition of IGF-1R signalling may be particularly effective among patients with non-visceral metastases [7].

Xentuzumab is a humanised monoclonal antibody that binds to the IGF-1 and IGF-2 ligands and neutralises their proliferative activity [6]. Xentuzumab demonstrated acceptable tolerability and preliminary antitumour activity in two Phase I studies in patients with advanced solid tumours [8]. A subsequent randomised Phase II study assessed xentuzumab plus everolimus and exemestane in patients with HR-positive/HER2-negative advanced breast cancer. Of note, this trial recruited patients prior to the approval of CDK4/6 inhibitors in this breast cancer setting. While addition of xentuzumab did not prolong progression-free survival (PFS) in the overall patient population, there was evidence of PFS benefit in the subgroup of patients without visceral metastases (hazard ratio 0.21, 95% confidence interval [CI] 0.05–0.98) [9].

Here, we report results of a randomised Phase II trial (NCT03659136) that evaluated the addition of xentuzumab to everolimus and exemestane in patients with HR-positive/HER2-negative advanced breast cancer with non-visceral disease who had received prior endocrine therapy with or without CDK4/6 inhibitors.

## Methods

### Study design and patients

This was a double-blind, randomised, placebo-controlled, Phase II trial, with potential seamless expansion to a confirmatory Phase III trial. Enrolment occurred between 15 May 2019 and 16 April 2021. Eligible patients were adult females diagnosed with histologically and centrally confirmed HR-positive/HER2-negative advanced or metastatic breast cancer not deemed amenable to curative surgery or radiation therapy. Patients were premenopausal receiving ovarian suppression or postmenopausal and must have experienced disease recurrence ≤ 12 months after completion of adjuvant endocrine therapy or had disease progression ≤ 1 month after the end of prior endocrine therapy for advanced breast cancer. Patients had to have at least one measurable non-visceral lesion per Response Evaluation Criteria in Solid Tumors (RECIST) v1.1 in either lymph nodes, soft tissue, skin and/or at least one measurable non-visceral bone lesion and/or at least one non-measurable bone lesion. In addition, patients should have Eastern Cooperative Oncology Group performance status of 0 or 1, fasting glucose < 8.9 mmol/L and HbA1c < 8.0%, and adequate organ function.

Key exclusion criteria were: evidence of visceral metastasis at screening; prior treatment with agents targeting the IGF, AKT or mTOR pathways or prior exemestane (prior treatment with a PI3K inhibitor, however, was not an exclusion criterion [10, 11]); more than one prior line of chemotherapy for HR-positive/HER2-negative metastatic breast cancer; more than one prior treatment line with a CDK4/6 inhibitor; major surgery within 4 weeks prior to randomisation; radiotherapy within 4 weeks prior to the start of study treatment; history or evidence of brain metastases; leptomeningeal carcinomatosis; or pre-existing interstitial lung disease.

### Treatment

Patients were randomised 1:1 to receive an intravenous infusion of xentuzumab (1000 mg/week) or placebo in combination with everolimus (10 mg/day orally) and exemestane (25 mg/day orally). Randomisation was stratified by the presence of baseline bone-only metastasis (yes vs no) and prior CDK4/6 inhibitor treatment (yes vs no). Dose reduction of xentuzumab was not permitted; however, treatment could be paused for up to 14 days for resolution of adverse events (AEs). Everolimus and exemestane treatment was administered in accordance with their respective Summary of Product Characteristics, Monograph or Prescribing Information (allowing dose interruption and adjustment for everolimus). Treatment continued until disease progression, unacceptable toxicity or other reasons requiring treatment discontinuation.

### Endpoints and assessments

The primary endpoint was PFS per independent review, defined as the time from randomisation until progressive disease (PD) according to RECIST v1.1 or death from any cause.

Secondary endpoints were: overall survival (OS; time from randomisation to death from any cause), disease control (best overall response of either complete response [CR], partial response [PR], stable disease [SD] or non-CR/non-PD; SD and non-CR/non-PD must have been observed until at least the Week 24 tumour assessment), duration of disease control, objective response (defined as best overall response of CR or PR), and time to pain progression or increase in pain treatment.

Tumours were assessed by computed tomography (CT) scans of the chest, abdomen and pelvis, a brain CT or magnetic resonance imaging, and a bone scan at baseline. If bone lesions were already known or confirmed at screening, bone scans had to be performed with every other CT scan and with an additional bone scan at Week 8. Assessments were performed every 8 weeks up to Week 80 (and every 12 weeks thereafter).

Tumour response and progression was evaluated per RECIST v1.1 in combination with MD Anderson criteria for patients with target and/or non-target bone lesions [12]. Response and progression were assessed by central independent review and by the investigator (independent assessments were considered primary; clinical decisions were based on investigator assessment).

Increases in pain treatment were measured based on the Analgesic Quantification Algorithm (AQA) and pain was measured with the Brief Pain Inventory – Short Form (BPI–SF). The BPI-SF is a nine-item, self-administered questionnaire to evaluate the severity of pain and its impact on daily functioning using a 10-point scale. A difference of two points is considered clinically meaningful [13].

Time to pain progression or increase in pain treatment was defined as the time from randomisation until the earliest of: a ≥ 2 point increase from baseline in the BPI-SF item 3 (worst pain), without a decrease (of ≥ 1 point) from baseline analgesics use (via the AQA), or a ≥ 2 point increase from baseline in the ADA, or death. Safety was assessed by the incidence and severity of AEs, graded according to National Cancer Institute Common Terminology Criteria for Adverse Events v5.0.

### Statistical analysis

Assuming a median PFS of 9.9 months in the placebo group based on the subgroup analysis of the BOLERO-2 study for those without visceral disease [14], and 19.8 months in the xentuzumab group based on the previous Phase II study (corresponding to a hazard ratio of 0.50) [9], it was calculated that 40 PFS events from a sample of 80 patients would be required for the Phase II part. This assumed approximately 20% of patients would discontinue the trial without evidence of disease progression or would be assessed as non-PD by independent assessment when the investigator assigned disease progression.

The primary analysis of PFS was planned to be conducted after 40 PFS events had occurred per independent review. Subgroup analyses of PFS were undertaken by independent review and supportive investigator assessment. Key prespecified subgroups were: presence of baseline bone-only metastases (yes vs no), prior CDK4/6 inhibitor treatment (yes vs no) and menopause status (pre vs post). A stratified Cox proportional hazards model was used to estimate the hazard ratio and its asymptotic two-sided 95% Wald CI between the two treatment groups (with a hazard ratio less than one favouring xentuzumab in combination with everolimus and exemestane). The primary efficacy analyses were performed on the randomised set (all randomised patients) and safety analyses were based on the treated set (all patients treated with at least one dose of any study treatment). As the trial was stopped early, no confirmatory statistical testing was possible, so the p-values are exploratory.

## Results

### Patients and treatment

**Fig. 1:**
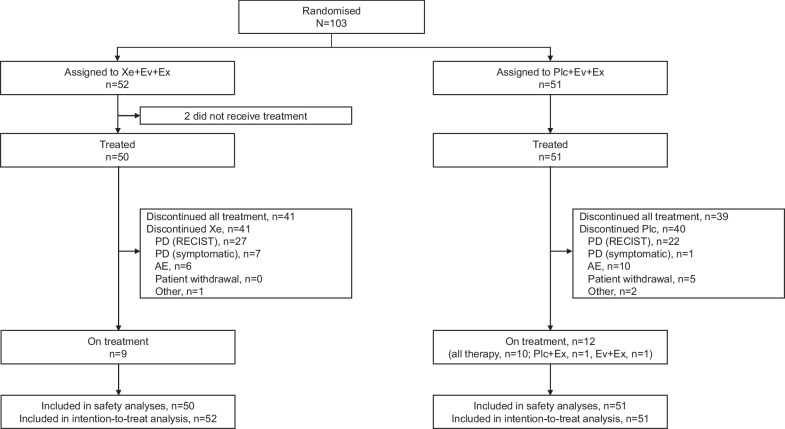
Patient disposition. AE, adverse event; Ev, everolimus; Ex, exemestane; PD, progressive disease; Plc, placebo; RECIST, Response Evaluation Criteria in Solid Tumors; Xe, xentuzumab

The trial was conducted in 53 centres in 11 countries (Australia, Belgium, Canada, France, Germany, Greece, Italy, Portugal, Spain, UK and USA). A total of 103 patients were randomised (52 to the xentuzumab arm and 51 to the placebo arm). Two patients assigned to the xentuzumab group were not treated (Fig. 1)*.* Most patients were postmenopausal (94.2%) and had bone-only metastases at screening (68.0%; Table 1). Approximately 75% of patients had received a prior CDK4/6 inhibitor. At data cut-off (September 2021), 41/50 patients and 39/51 patients had discontinued treatment in the xentuzumab and placebo arms, respectively. Most patients discontinued due to PD (Fig. 1). Median duration of treatment was similar across treatment arms (6.8 months in the xentuzumab group and 6.2 months in the placebo group).

**Table 1:** Patient demographic and baseline characteristics

	Xe1000 + Ev10 + Ex25 n = 52	Plc + Ev10 + Ex25 n = 51	Total N = 103
Median age, years (range)	60.5 (29–84)	59.0 (41–78)	60.0 (29–84)
Race, n (%)			
Black or African American	0	1 (2.0)	1 (1.0)
Asian	1 (1.9)	0	1 (1.0)
Native Hawaiian or other Pacific Islander	1 (1.9)	0	1 (1.0)
White	44 (84.6)	41 (80.4)	85 (82.5)
Missing	6 (11.5)	9 (17.6)	15 (14.6)
ECOG PS, n (%)			
0	38 (73.1)	30 (58.8)	68 (66.0)
1	14 (26.9)	21 (41.2)	35 (34.0)
Menopausal status, n (%)			
Premenopausal	1 (1.9)	4 (7.8)	5 (4.9)
Postmenopausal	50 (96.2)	47 (92.2)	97 (94.2)
Missing	1 (1.9)^a^	0	1 (1.0)^a^
Bone-only metastases at screening, n (%)			
No	13 (25.0)	19 (37.3)	32 (31.1)
Yes	38 (73.1)	32 (62.7)	70 (68.0)
Missing	1 (1.9)^a^	0	1 (1.0)^a^
Previous systemic chemotherapy in metastatic setting, n (%)	6 (11.5)	3 (5.9)	9 (8.7)
Previous fulvestrant treatment,^b^ n (%)	26 (50.0)	19 (37.3)	45 (43.7)
Previous CDK4/6 inhibitor,^b^ n (%)	39 (75.0)	39 (76.5)	78 (75.7)
Endocrine resistance,^c^ n (%)			
Primary	15 (28.8)	12 (23.5)	27 (26.2)
Secondary	37 (71.2)	39 (76.5)	76 (73.8)

CDK4/6, cyclin-dependent kinase 4/6; ECOG PS, Eastern Cooperative Oncology Group performance status; Ev, everolimus; Ex, exemestane; Plc, placebo; Xe, xentuzumab

^a^Missing data relates to one patient who was randomised but not treated; ^b^In the adjuvant, neoadjuvant or metastatic setting; ^c^Primary resistance defined as relapse within 24 months of starting neoadjuvant or adjuvant treatment or progressive disease within 6 months of starting metastatic treatment. Secondary resistance defined as relapse ≥ 24 months on neoadjuvant or adjuvant treatment, or < 12 months after completion, or progressive disease ≥ 6 months after starting metastatic treatment

### Efficacy

Due to discordance between investigator and independent assessment of PFS, it was determined that 40 PFS events based on independent assessment would not occur. As such, the primary analysis was conducted after approximately 40 PFS events per investigator assessment; nevertheless, primary analysis was still based on independent assessment.

**Fig. 2:**
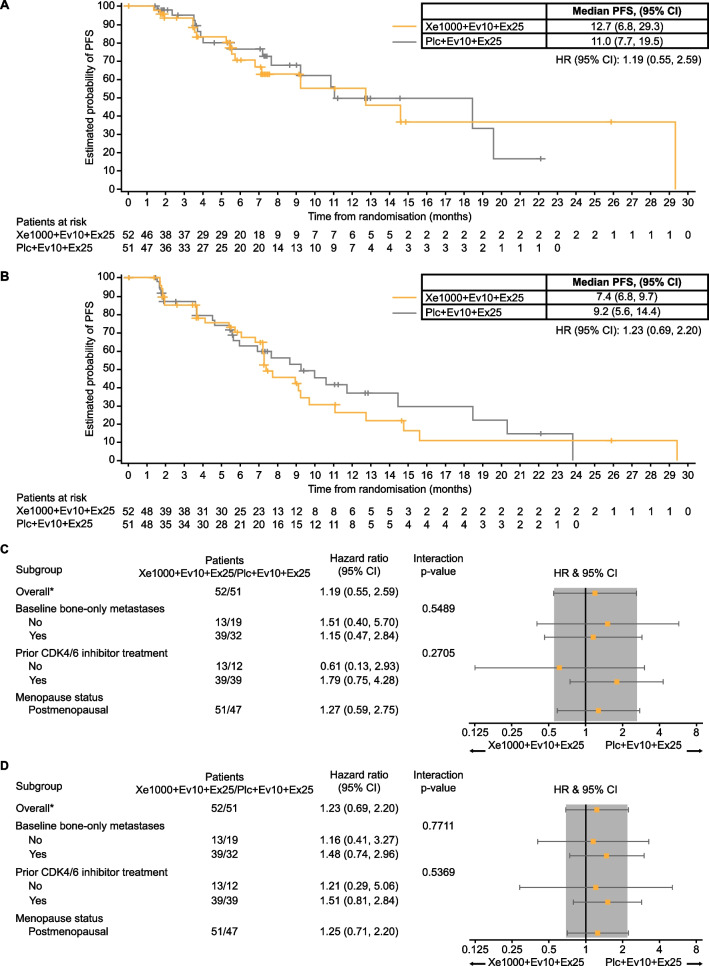
Progression-free survival for xentuzumab plus everolimus and exemestane versus everolimus and exemestane. **A** According to independent assessment. **B** According to investigator assessment. **C** Subgroup analysis of progression-free survival by independent assessment. **D** Subgroup analysis of progression-free survival by investigator assessment. CI, confidence interval; Ev, everolimus; Ex, exemestane; HR, hazard ratio; Plc, placebo; Xe, xentuzumab

At the time of analysis, 17 patients (32.7%) in the xentuzumab arm and 15 (29.4%) in the placebo arm had experienced a PFS event per independent assessment. Median PFS was 12.7 (95% CI 6.8–29.3) months with xentuzumab and 11.0 (7.7–19.5) months with placebo (hazard ratio 1.19; 95% CI 0.55–2.59; p = 0.6534; Fig. 2A). Per investigator assessment, 30 patients (57.7%) in the xentuzumab arm and 26 (51.0%) in the placebo arm had experienced a PFS event. Median PFS was 7.4 (6.8–9.7) months with xentuzumab and 9.2 (5.6–14.4) months with placebo (hazard ratio 1.23; 95% CI 0.69–2.20; p = 0.4800; Fig. 2B).

Subgroup analyses of PFS were generally consistent with the overall analyses with no statistically significant differences (Figs. 2C and 2D). The only subgroup that demonstrated numerically higher median PFS in the xentuzumab arm than in the placebo arm per independent assessment was patients with no prior CDK4/6 inhibitor exposure (n = 25; 29.3 months vs 18.4 months; Additional file 1: Table S1). In both arms, patients who had received a CDK4/6 inhibitor had shorter PFS than patients who were CDK4/6 inhibitor-naïve (Additional file 1: Table S1). These differences were not as pronounced per investigator assessment (Additional file 1: Table S1).

Five (9.6%) patients in the xentuzumab group and eight (15.7%) in the placebo group had died at data cut-off. OS data were immature and median OS was not calculable in either treatment group. Preliminary analysis indicated that there was no statistical difference in OS between the two arms (p = 0.1983).

Per independent review, disease control was achieved in 29 (55.8%) patients in the xentuzumab arm versus 25 (49.0%) patients in the placebo arm (odds ratio [OR] 1.31; 95% CI 0.60–2.86). Median duration of disease control was 14.6 and 18.4 months, respectively. Per investigator assessment, disease control rate was 57.7% in the xentuzumab arm and 51.0% in the placebo arm (OR 1.31; 95% CI 0.60–2.87). In line with the predominantly non-measurable bone-only disease setting, the objective response rate was 11.5% and 9.8% in the xentuzumab and placebo arms, respectively, per independent assessment (OR 1.20; 95% CI 0.34–4.43; p = 0.7759).

Time to pain progression or increase in pain treatment was not significantly different between the treatment groups (median of 5.6 versus 3.0 months for xentuzumab and placebo, respectively; hazard ratio 0.97; 95% CI 0.54–1.76; p = 0.9279).

### Safety

A total of 48 (96.0%) and 50 (98.0%) patients experienced AEs in the xentuzumab and placebo arms, respectively. The most common AEs were diarrhoea, fatigue and headache (Table 2). There was one grade 5 AE (pneumonia in the placebo arm) which was not considered related to treatment. The most common treatment-related AEs (TRAEs; deemed related to any of the study treatments) were diarrhoea, fatigue and mucosal inflammation (Additional file 1: Table S2). There was one grade 4 TRAE (acute kidney injury in the placebo arm); all other TRAEs were grade 3 or lower. The incidence of hyperglycaemia was similar in the xentuzumab and placebo arms (all grades: 11/50 [22.0%] versus 13/51 [25.5%]; grade 3: 1/50 [2.0%] versus 3/51 [5.9%], respectively); there were no incidences of grade 4 or 5 hyperglycaemia in either arm.

**Table 2:** Most common treatment-emergent adverse events (occurring in > 15% of patients in either treatment arm)

	Xe1000 + Ev10 + Ex25 (n = 50)		Plc + Ev10 + Ex25 (n = 51)	
	All grades	Grade ≥ 3	All grades	Grade ≥ 3
Patients with any AE, n (%)	48 (96.0)	28 (56.0)	50 (98.0)	28 (54.9)
Diarrhoea	28 (56.0)	3 (6.0)	17 (33.3)	0
Fatigue	22 (44.0)	4 (8.0)	17 (33.3)	1 (2.0)
Headache	20 (40.0)	0	11 (21.6)	0
Nausea	18 (36.0)	2 (4.0)	14 (27.5)	0
Decreased appetite	18 (36.0)	1 (2.0)	17 (33.3)	0
Arthralgia	16 (32.0)	1 (2.0)	16 (31.4)	1 (2.0)
Mucosal inflammation	15 (30.0)	0	16 (31.4)	3 (5.9)
Epistaxis	15 (30.0)	0	7 (13.7)	0
Stomatitis	14 (28.0)	1 (2.0)	15 (29.4)	4 (7.8)
Rash	13 (26.0)	0	9 (17.6)	0
Muscle spasms	12 (24.0)	0	5 (9.8)	0
Cough	12 (24.0)	0	8 (15.7)	0
Thrombocytopenia	11 (22.0)	0	1 (2.0)	0
Hyperglycaemia	11 (22.0)	1 (2.0)	13 (25.5)	3 (5.9)
Anaemia	10 (20.0)	1 (2.0)	13 (25.5)	1 (2.0)
Dysgeusia	10 (20.0)	0	5 (9.8)	0
Asthenia	10 (20.0)	1 (2.0)	13 (25.5)	1 (2.0)
Vomiting	10 (20.0)	1 (2.0)	8 (15.7)	0
Platelet count increased	9 (18.0)	3 (6.0)	5 (9.8)	0
Neutropenia	9 (18.0)	2 (4.0)	3 (5.9)	1 (2.0)
Pruritus	9 (18.0)	1 (2.0)	9 (17.6)	0
Urinary tract infection	9 (18.0)	2 (4.0)	9 (17.6)	0
Platelet count decreased	9 (18.0)	3 (6.0)	5 (9.8)	0
Upper abdominal pain	8 (16.0)	1 (2.0)	3 (5.9)	0
Pyrexia	8 (16.0)	1 (2.0)	9 (17.6)	0
ALT increased	8 (16.0)	0	7 (13.7)	2 (3.9)
Dizziness	8 (16.0)	0	4 (7.8)	1 (2.0)
Dyspnoea	7 (14.0)	0	8 (15.7)	1 (2.0)
Hypertension	6 (12.0)	0	8 (15.7)	1 (2.0)
Pneumonitis	5 (10.0)	0	13 (25.5)	3 (5.9)
Constipation	5 (10.0)	0	8 (15.7)	0
AST increased	5 (10.0)	1 (2.0)	8 (15.7)	2 (3.9)
Peripheral oedema	3 (6.0)	0	9 (17.6)	1 (2.0)
Back pain	3 (6.0)	0	9 (17.6)	0

AE, adverse event; ALT, alanine aminotransferase; AST, aspartate aminotransferase; Ev, everolimus; Ex, exemestane; Plc, placebo; Xe, xentuzumab

Seven (14.0%) patients had AEs leading to discontinuation of xentuzumab and 10 (19.6%) had AEs leading to discontinuation of placebo. AEs leading to discontinuation of exemestane were similar in both treatment arms (five [10.0%] in the xentuzumab arm and seven [13.7%] in the placebo arm). AEs leading to discontinuation or dose reduction of everolimus were numerically higher in the placebo arm than in the xentuzumab arm (15 [29.4%] versus eight [16.0%] and 17 [33.3%] versus 13 [26.0%], respectively). There was one dose interruption for a grade 1 infusion-related reaction in the xentuzumab arm.

Serious AEs (SAEs) were reported in 13 (26.0%) patients in the xentuzumab arm and 17 (33.3%) patients in the placebo arm. The most common SAEs in the xentuzumab and placebo arms were pneumonitis (one [2.0%] and three [5.9%], respectively), hyperglycaemia (two [4.0%] and one [2.0%]), angioedema (three [6.0%] and 0) and COVID-19 pneumonia (0 and two [3.9%]). There were three infusion-related reactions during the trial (two in the xentuzumab group and one in the placebo group).

The COVID pandemic occurred while this study was being conducted and affected recruitment with a hold on recruitment between March–May 2020. The frequency of COVID, COVID pneumonia and suspected COVID were 0.0%/9.8%, 0.0%/3.9% and 2.0%/0.0% in the xentuzumab and placebo arms, respectively. One patient in each arm discontinued treatment because of COVID, but there were no COVID-related deaths.

## Discussion

In this study, addition of xentuzumab to everolimus and exemestane did not confer PFS benefit versus placebo in patients with HR-positive/HER2-negative metastatic breast cancer and non-visceral disease. There were no clear differences in safety profile between the treatment arms and no new safety signals were observed; overall rates of AEs, TRAEs and SAEs were similar across treatment groups.

The IGF signalling pathway has long been recognised as a potential therapeutic target in many cancer types [15]. In particular, the IGF axis is thought to play a key role in the development of resistance to other cancer therapies by acting as an escape pathway [16]. Early strategies for targeting the IGF-1R included monoclonal antibodies and tyrosine kinase inhibitors (TKIs). While such agents showed potential activity as single agents, subsequent combination trials in a variety of tumour types were disappointing [17–21]. It was hypothesised that the anti-proliferative activity of the anti-IGF-1R antibodies may have been limited by compensatory signalling via another member of the IGF axis – insulin receptor (IR)-A. IGF-1R-targeted TKIs were limited by co-inhibition of IR-B, which regulates glucose uptake, and thus were associated with an increased risk of hyperglycaemia [16, 22]. It was anticipated that the different mechanism of action of xentuzumab – which neutralises the IGF-1 and -2 ligands – could overcome these limitations, by inhibiting proliferative signalling via IGF-1R and IR-A, without affecting IR-B signalling. Indeed, there was no evidence of increased risk of hyperglycaemia with xentuzumab versus placebo.

While markers of target engagement were not assessed in this study, previous studies and preclinical data have shown that xentuzumab effectively inhibits IGF signalling, with reductions in IGF bioactivity following xentuzumab administration [6, 8]. As such, we hypothesise that the lack of activity observed in this study indicates that either the IGF pathway is not the main driver of disease, or that other pathways are able to upregulate and compensate for inhibition. It has also been hypothesised that biomarkers could be used to identify patients who would specifically benefit from IGF-targeted treatment. However, future investigations are required to better understand and define which predictive biomarkers could be used. Additional research is also required to better understand the alternative pathways that could be compensating for the inhibition of IGF [23, 24].

Of note, median PFS in both arms of this trial was substantially longer than that observed in other recent trials undertaken in a similar setting, e.g. EMERALD (elacestrant versus endocrine monotherapy; median PFS 1.9–2.8 months) and VERONICA (venetoclax plus fulvestrant versus fulvestrant; median PFS 1.9–2.7 months) [25, 26]. In both studies, patients had received prior CDK4/6 inhibitor therapy. Potential explanations for the favourable PFS in this study include the fact that patients in this study had non-visceral metastases, the addition of everolimus to endocrine therapy in our patients, or the lower proportion of patients in this study with prior CDK4/6 exposure (75.7%). Recent findings have shown that the durations of response with everolimus regimens post-CDK4/6 inhibitor therapy are shorter than those observed in the pre-CDK4/6 era. This is likely due to the generally poorer outcomes in later treatment [2, 4, 27–30].

This trial was unblinded and analysed early due to high rates of discordance between independent and investigator assessment of PFS. In both arms, median PFS was shorter when assessed by investigators rather than independently. In most cases, discordance was due to the investigator identifying PD when the independent reviewer did not. This is not unexpected in this patient population in which many patients had non-measurable disease. While independent reviewers were provided skin lesion photographs, biopsy reports from new lesions and supportive imaging if available, investigators may have been using additional parameters (e.g. specific tumour markers) to assess progression which were not considered for independent analysis. Similar observations were made in the BOLERO-2 trial (median PFS for everolimus/exemestane vs placebo/exemestane was 6.9 months vs 2.8 months [investigator assessment] and 10.6 vs 4.1 months [independent assessment]) [2].

## Conclusions

Overall, while xentuzumab could be safely combined with everolimus and exemestane, the addition of xentuzumab did not improve PFS in patients with HR-positive, HER2-negative advanced breast cancer with non-visceral disease.

## Supplementary Information

**Additional file 1.** Supplementary Material.

## Data Availability

To ensure independent interpretation of clinical study results and enable authors to fulfill their role and obligations under the ICMJE criteria, Boehringer Ingelheim grants all external authors access to clinical study data pertinent to the development of the publication. In adherence with the Boehringer Ingelheim Policy on Transparency and Publication of Clinical Study Data, scientific and medical researchers can request access to clinical study data after publication of the primary manuscript in a peer-reviewed journal, regulatory activities are complete and other criteria are met. Researchers should use the https://vivli.org/ link to request access to study data and visit https://www.mystudywindow.com/msw/datasharing for further information.

## Abbreviations

AE: Adverse event
AQA: Analgesic Quantification Algorithm
BPI-SF: Brief Pain Inventory-Short Form
CDK: Cyclin dependent kinase
CI: Confidence interval
CR: Complete response
CT: Computed tomography
ECOG PS: Eastern Cooperative Oncology Group performance status
HER2: Human epidermal growth factor receptor 2
HR: Hormone receptor
IGF: Insulin-like growth factor
IGF-1R: Insulin-like growth factor type 1 receptor
mTOR: Mammalian target of rapamycin
OR: Objective response
OS: Overall survival
PD: Progressive disease
PFS: Progression-free survival
PR: Partial response
RECIST: Response Evaluation Criteria in Solid Tumors
SD: Stable disease
TRAE: Treatment-related adverse event
