# Supplementary material for: XENERA-1: a randomised double-blind Phase II trial of xentuzumab in combination with everolimus and exemestane versus everolimus and exemestane in patients with hormone receptor-positive/HER2-negative metastatic breast cancer and non-visceral disease
Source: Breast Cancer Res. 2023 Jun 12;25:67. doi: 10.1186/s13058-023-01649-w (PMC10258741; doi:10.1186/s13058-023-01649-w)
Supplement: Supplementary file 1 — Additional file 1. Supplementary Material. [file 13058_2023_1649_MOESM1_ESM.docx]

**Supplementary Material.**

**Supplementary Table S1.** Subgroup analysis of progression-free survival.

|  | **Xe1000+Ev10+Ex25 (n=50)** | | **Plc+Ev10+Ex25 (n=51)** | |  |  |
| --- | --- | --- | --- | --- | --- | --- |
| **Subgroup** | **n** | **Median PFS (IQR)** | **n** | **Median PFS (IQR)** | **Hazard ratio (95% CI)** | **p-value** |
| **Independent assessment** |  |  |  |  |  |  |
| Bone-only metastases |  |  |  |  |  |  |
| No | 13 | 5.6 (5.3–NC) | 19 | 18.4 (3.9–18.4) | 1.51 (0.40–5.70) | 0.5422 |
| Yes | 39 | 12.7 (6.8–29.3) | 32 | 11.0 (9.2–19.5) | 1.15 (0.47–2.84) | 0.7576 |
| Prior CDK4/6 inhibitor treatment |  |  |  |  |  |  |
| No | 13 | 29.3 (6.8–29.3) | 12 | 18.4 (9.2–19.5) | 0.61 (0.13–2.93) | 0.5328 |
| Yes | 39 | 9.2 (5.3–14.6) | 39 | 10.8 (5.5–NC) | 1.79 (0.75–4.28) | 0.1918 |
| Postmenopausal | 51 | 12.7 (5.6–29.3) | 47 | 11.0 (5.5–19.5) | 1.27 (0.59–2.75) | 0.5376 |
| **Investigator assessment** |  |  |  |  |  |  |
| Bone-only metastases |  |  |  |  |  |  |
| No | 13 | 7.4 (3.7–8.9) | 19 | 5.6 (3.6–18.4) | 1.16 (0.41–3.27) | 0.7850 |
| Yes | 39 | 7.7 (5.7–14.8) | 32 | 11.7 (5.9–20.3) | 1.48 (0.74–2.96) | 0.2688 |
| Prior CDK4/6 inhibitor treatment |  |  |  |  |  |  |
| No | 13 | 11.1 (6.0–14.8) | 12 | 9.2 (5.6–18.4) | 1.21 (0.29–5.06) | 0.7917 |
| Yes | 39 | 7.3 (3.7–9.2) | 39 | 10.0 (4.6–14.4) | 1.51 (0.81–2.84) | 0.1961 |
| Postmenopausal | 51 | 7.4 (4.1–12.7) | 47 | 8.6 (4.5–18.4) | 1.25 (0.71–2.20) | 0.4342 |

CI, confidence interval; Ev, everolimus; Ex, exemestane; IQR, interquartile range; PFS, progression-free survival; Plc, placebo; Xe, xentuzumab.

**Supplementary Table S2.** Most common treatment-related adverse events (occurring in ≥10% of patients in either treatment arm)

|  | **Xe1000+Ev10+Ex25 (n=50)** | | | **Plc+Ev10+Ex25 (n=51)** | | | |
| --- | --- | --- | --- | --- | --- | --- | --- |
|  | **All grades** | | **Grade ≥3^a^** | | **All grades** | | **Grade ≥3^a^** |
| Patients with any TRAE, n (%) | 48 (96.0) | | 22 (44.0) | | 48 (94.1) | | 23 (45.1) |
| Diarrhoea | 17 (34.0) | | 1 (2.0) | | 9 (17.6) | | 0 |
| Fatigue | 17 (34.0) | | 3 (6.0) | | 8 (15.7) | | 0 |
| Mucosal inflammation | 15 (30.0) | | 0 | | 16 (31.4) | | 3 (5.9) |
| Stomatitis | 13 (26.0) | | 1 (2.0) | | 14 (27.5) | | 4 (7.8) |
| Decreased appetite | 13 (26.0) | | 1 (2.0) | | 11 (21.6) | | 0 |
| Epistaxis | 11 (22.0) | | 0 | | 4 (7.8) | | 0 |
| Anaemia | 10 (20.0) | | 1 (2.0) | | 8 (15.7) | | 1 (2.0) |
| Thrombocytopenia | 10 (20.0) | | 0 | | 1 (2.0) | | 0 |
| Hyperglycaemia | 10 (20.0) | | 1 (2.0) | | 11 (21.6) | | 3 (5.9) |
| Nausea | 9 (18.0) | | 0 | | 7 (13.7) | | 0 |
| Rash | 9 (18.0) | | 0 | | 8 (15.7) | | 0 |
| Dysgeusia | 8 (16.0) | | 0 | | 3 (5.9) | | 0 |
| Platelet count increased | 8 (16.0) | | 3 (6.0) | | 4 (7.8) | | 0 |
| Neutropenia | 7 (14.0) | 1 (2.0) | | 2 (3.9) | | 1 (2.0) | |
| Asthenia | 7 (14.0) | 0 | | 7 (13.7) | | 0 | |
| Arthralgia | 7 (14.0) | 0 | | 4 (7.8) | | 0 | |
| Headache | 6 (12.0) | 0 | | 2 (3.9) | | 0 | |
| Pruritus | 6 (12.0) | 0 | | 8 (15.7) | | 0 | |
| ALT increased | 5 (10.0) | 0 | | 4 (7.8) | | 2 (3.9) | |
| Hypertriglyceridemia | 5 (10.0) | 0 | | 5 (9.8) | | 1 (2.0) | |
| Myalgia | 5 (10.0) | 0 | | 2 (3.9) | | 0 | |
| Muscle spasms | 5 (10.0) | 0 | | 1 (2.0) | | 0 | |
| Pneumonitis | 5 (10.0) | 0 | | 13 (25.5) | | 3 (5.9) | |
| Dry skin | 5 (10.0) | 0 | | 2 (3.9) | | 0 | |
| Neutrophil count decreased | 4 (8.0) | 1 (2.0) | | 6 (11.8) | | 1 (2.0) | |

^a^There was one grade 4 TRAE: acute kidney injury in a patient in the placebo arm

ALT, alanine aminotransferase; Ev, everolimus; Ex, exemestane; Plc, placebo; TRAE, treatment-related AE; Xe, xentuzumab.
